# Selective Neuromodulation of the Vagus Nerve

**DOI:** 10.3389/fnins.2021.685872

**Published:** 2021-05-24

**Authors:** Adam Fitchett, Svetlana Mastitskaya, Kirill Aristovich

**Affiliations:** Department of Medical Physics and Biomedical Engineering, University College London, London, United Kingdom

**Keywords:** vagus nerve, fascicular anatomy, electrical stimulation, neuromodulation, fiber-specificity, spatial specificity

## Abstract

Vagus nerve stimulation (VNS) is an effective technique for the treatment of refractory epilepsy and shows potential for the treatment of a range of other serious conditions. However, until now stimulation has generally been supramaximal and non-selective, resulting in a range of side effects. Selective VNS (sVNS) aims to mitigate this by targeting specific fiber types within the nerve to produce functionally specific effects. In recent years, several key paradigms of sVNS have been developed—spatially selective, fiber-selective, anodal block, neural titration, and kilohertz electrical stimulation block—as well as various stimulation pulse parameters and electrode array geometries. sVNS can significantly reduce the severity of side effects, and in some cases increase efficacy of the treatment. While most studies have focused on fiber-selective sVNS, spatially selective sVNS has demonstrated comparable mitigation of side-effects. It has the potential to achieve greater specificity and provide crucial information about vagal nerve physiology. Anodal block achieves strong side-effect mitigation too, but is much less specific than fiber- and spatially selective paradigms. The major hurdle to achieving better selectivity of VNS is a limited knowledge of functional anatomical organization of vagus nerve. It is also crucial to optimize electrode array geometry and pulse shape, as well as expand the applications of sVNS beyond the current focus on cardiovascular disease.

## Introduction

The vagus nerve (VN) is one of the most promising targets for neuromodulation. The discovery in the 1980s that VN stimulation (VNS) can stop seizures in dogs lead to VNS for epilepsy treatment, with the first VN stimulators approved by the United States Federal Drug Administration in 1997 (Krahl, 2012). By 2018, over 100,000 patients had been implanted with VNS devices (Purser et al., 2018). Since the 1990s, evidence of a role for the VN in regulating diverse physiological functions has sparked interest in VNS beyond epilepsy treatment; VNS has been investigated for addressing treatment-resistant depression, cardiovascular disease, sepsis, chronic pain, obesity, diabetes, lung injury, stroke, traumatic brain injury and arthritis (Johnson and Wilson, 2018). This interest has driven the continuous development of better VNS devices and stimulation techniques.

A major motivation to optimize the implementation of VNS has been the prevalence of side effects (Noller et al., 2019), including bradycardia, bradypnea, apnea, indigestion, throat and tonsil pain, cough, hoarseness, nausea and vomiting, headache, diaphragmatic flutter and paresthesia (Ben-Menachem, 2001, 2002). These side effects result primarily from the common practice of stimulating the whole VN, as opposed to selectively stimulating only the parts responsible for modulating a given function (Plachta et al., 2014; Aristovich et al., 2021).

This review presents a brief overview of major clinical applications of VNS with regard to known anatomy and physiological functions of the VN, followed by in-depth discussion of major paradigms for selective VNS (sVNS) and the advantages of each paradigm. It is a focused review which attempts to cover the recent studies on sVNS. It provides an assessment of the future clinical applicability of sVNS and discusses what recent attempts to achieve selective activation of nerve fibers have revealed about VN physiology.

### Anatomy and Functions of the Vagus Nerve

**FIGURE 1:**
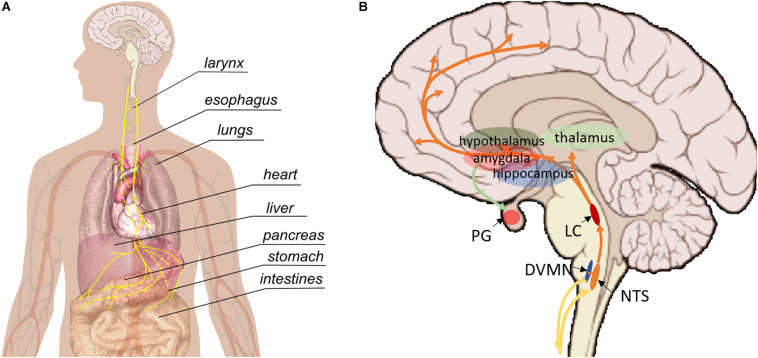
Pathways involved in vagus nerve stimulation. **(A)** Peripherally, the vagus nerve provides afferent and efferent innervation of the majority of visceral organs. **(B)** Central regions that are impacted by vagus nerve stimulation. NTS, nucleus tractus solitarii; DVMN, dorsal motor nucleus of the VN; LC, locus coeruleus, PG, pituitary gland.

The VN (whose name means “wandering”) is the longest nerve in the autonomic nervous system, projecting from the brain to a number of organs in the thorax and abdomen including the heart, lungs, larynx, pharynx, stomach, spleen, pancreas, liver, intestines, and ovaries (Figure 1A; Thompson et al., 2019). There are two VNs (left and right), but convention is to refer to the VN in the singular, even though there are some functional differences between the two VNs; most importantly, the right VN innervates the sinoatrial node of the heart whereas the left VN innervates the atrioventricular node. Despite extensive research over the last century, the functional fascicular anatomy of this complex nerve remains poorly understood (Thompson et al., 2020).

The VN contains both efferent and afferent fibers. The afferent fibers make up the vast majority (up to 90%) and relay interoceptive information from various organs to the brain and spinal cord; the remainder are parasympathetic efferents that allow the VN to influence the activity of innervated organs (Thompson et al., 2019). The VN also projects to areas within the brain and central nervous system (CNS), including the nucleus tractus solitarius (NTS), locus coeruleus (LC), thalamus, hippocampus, amygdala and other regions (Figure 1B; Thompson et al., 2019). Parasympathetic efferents originate in the dorsal motor nucleus and nucleus ambiguous. Sensory afferents within the cervical VN have their cell bodies in the nodose ganglia and extend their central projections to the NTS (Thompson et al., 2019).

Like all large peripheral nerves, the VN contains a mixture of different types of nerve fibers, which are organized into bundles (fascicles). The fibers vary in diameter and conduction velocity, with Aα fibers the largest and fastest (diameter 13–20 μm, conduction velocity 80–120 ms^–1^), C fibers the smallest and slowest (0.2–1.5 μm, 0.5–2 ms^–1^), and Aβ, Aγ, Aδ, and B fibers intermediate (Whitwam, 1976; Kandel et al., 2020). In humans, the VN at the cervical level typically contains between 5–8 fascicles, but individual variations have been documented at 1–21 per side) (Hammer et al., 2018). It is not definitely known whether each fascicle contains only one type of fiber (afferent or efferent) or both, or whether the fascicles are somatotopically arranged (i.e., arranged according to end-effect organ). Although, there is evidence for the latter and against the former (Settell et al., 2020).

To avoid off-target effects and improve overall efficacy of VNS, it is necessary to selectively stimulate fibers with known anatomical projection within the trunk of the VN (Thompson et al., 2019). On one hand, organ-specific branching of the VN, somatotopic organization of the cell bodies of vagal neurons in the brainstem nuclei, and clear evidence that VNS can elicit functionally specific physiological effects (Aristovich et al., 2021) have strengthened the belief that VN fibers are grouped somatotopically. On the other hand, evidence in the pig suggests a “bimodal” organization, in which motor and sensory fibers form two spatially distinct groups (Settell et al., 2020). It is possible that fibers are organized both bimodally and somatotopically. It is also important to bear in mind that anatomical variation of the VN exists between species, and so data from experimental animals do not necessarily translate to humans (Thompson et al., 2019). For example, there is evidence for somatotopic organization of the pseudounipolar cell bodies of sensory afferents in nodose ganglia. However, more recent research has suggested that this organization may only be present in pigs but not in humans (Settell et al., 2021). Large differences also exist in the diameter of the VN between species; this should be considered when evaluating the translational potential of VNS techniques demonstrated in small animals (such as rodents).

### Side Effects of Non-selective VNS

During nerve stimulation, fibers are activated in order of size from the largest (A fibers) to the smallest (C fibers). The majority of side effects of nsVNS that limit its therapeutic efficacy (throat and tonsil pain, hoarseness) are associated with the activation of large A fibers innervating the mucosa and muscles of larynx and pharynx (Gold et al., 2016). Cough, another common side effect of VNS, is a reflex response to activation of rapidly adapting pulmonary stretch receptors (Aδ myelinated fibers in pulmonary epithelium) (Kubin et al., 2006).

Acute apnea and bradypnea result from the Hering-Breuer inflation reflex caused by stimulation of pulmonary A-fiber afferents innervating the slowly adaptive pulmonary stretch receptors present in the smooth muscles of the airways (Kubin et al., 2006). Cardioinhibitory action of VNS is attributed to stimulation of efferent myelinated B fibers (Qing et al., 2018). Bradycardia may also result from activation of the sinoatrial node when stimulating the right VN. For this reason, VNS is generally applied on the left side (Krahl, 2012). It is evident that electrical stimulation applied to the cervical VN preferentially activates large motor and sensory fibers (Aα and Aβ fibers) because they have a lower activation threshold than smaller efferent fibers (Ardell et al., 2017), therefore, simply altering parameters such as frequency and amplitude is not sufficient to alleviate these side effects, and may lead to a reduction in efficacy (Handforth et al., 1998; Ardell et al., 2017; Aristovich et al., 2021). Aα motor efferents projecting to the larynx also have low activation threshold. It is interesting to note that many papers do not specify the parameters used for VNS (Kwan et al., 2016). Systematic reporting of parameters in studies where VNS is used would greatly assist in optimizing those parameters for the reduction of side effects.

Unintentional activation of low-threshold motor nerve fibers during nsVNS could also be attributed to sub-optimal insulation of the electrode array. The current may leak out of the insulation, spreading to and activating any nearby fibers with a sufficiently low activation threshold (Nicolai et al., 2020). It is important to note that the risk of the current leak would be magnified by the use of more complex circuits, which is the case in sVNS where multiple current sources are usually required. This should be taken into account when designing the devices for sVNS.

## Applications of VNS

Stimulation of the whole left cervical VN is an FDA-approved treatment for focal epilepsy and treatment-resistant depression (Krahl and Clark, 2012; Lv et al., 2019). Other clinical applications that have been explored for VNS include generalized seizures, cardiovascular disease, inflammation, obesity, chronic pain, respiratory disease, traumatic brain injury, stroke, post-traumatic stress disorder (Johnson and Wilson, 2018). Of these, cardiovascular applications have proven of greatest interest in the development of sVNS, due to the need to selectively target smaller diameter vagal fibers innervating the heart.

### Heart Failure

Autonomic nervous system dysfunction, due to excessive sympatho-excitation and withdrawal of parasympathetic (vagal) tone, is a key mechanism of heart failure (Binkley et al., 1991; Floras and Ponikowski, 2015). VNS increased survival, slowed down the progression of myocardial remodeling, and improved ventricular function in numerous experimental models of heart failure (Li et al., 2004; Agarwal et al., 2016) as well as in some clinical studies (De Ferrari et al., 2010; Premchand et al., 2014). Moderate electrical stimulation (up to 2 mA) applied to the cervical VN preferentially activates afferent sensory fibers, which have a lower activation threshold than efferent fibers. This leads to a reflex low-level sympatho-excitation and increased heart rate (HR) (Ardell et al., 2017). Aggressive stimulation (>2.5 mA) could recruit efferent fibers responsible for vagally mediated lowering of the HR, but this would cause significant side effects, including dysphonia, neck pain, and cough (Zannad et al., 2014).

### Ischemia-Reperfusion Injury

Myocardial ischemia-reperfusion injury (IR injury) refers to myocardial damage caused by blood supply returning to myocardial tissue following ischemia. It is frequently triggered by clinical therapies such as thrombolytic therapy or percutaneous transluminal coronary intervention (PCI). Preclinical studies in rats have shown that – following IR injury – VNS decreases infarct size, inflammation and incidence of ischemia-induced arrhythmias, oxidative stress, and apoptosis in cardiomyocytes (Mioni et al., 2005; Calvillo et al., 2011). These effects are predominantly mediated by efferent vagal fibers (Mastitskaya et al., 2012; Nuntaphum et al., 2018). There are no clinical data on cervical VNS in acute myocardial injuries.

### Arrhythmia

VNS has successfully been used to manage both atrial and ventricular arrhythmias in preclinical and clinical studies (Li and Yang, 2009). VNS administered at a voltage below the bradycardia threshold significantly increases the effective refractory period, which suppresses atrial fibrillation (Li and Yang, 2009). It would be interesting to explore the comparative efficacy of potential sVNS techniques to manage arrhythmia without the risk of unintentional bradycardia.

### Focal Epilepsy

Epilepsy is a chronic neurological disorder characterized by episodes of aberrant synchronous neural activity, also known as seizures. Seizures can result in loss of consciousness, loss of motor coordination and other neurological symptoms (Scheffer et al., 2017). Epilepsy is generally treated with anti-epileptic drugs, but up to 30% of cases do not respond to medication (Moshé et al., 2015). For these refractory epilepsy patients, surgical resection of the epileptogenic zone may be necessary. However, this cannot be carried out in 50% of patients and is ineffective in 30% of the remaining ones (Neligan et al., 2012). VNS of the left cervical VN has emerged as a safe and reliable means of treating such patients. It is believed that solely afferent fibers are involved in the mechanisms of VNS therapeutic effects for epilepsy, because epilepsy is primarily a disorder of the brain. Accordingly, an optimal seizure-suppressive sVNS would avoid activation of efferent fibers projecting to the heart, lungs and other organs in the torso. If the internal anatomy of the VN is functionally organized, it would also be desirable to locate and selectively activate the fascicles responsible for seizure-suppression (Thompson et al., 2019). It is not known which VN fascicles, if any, project to implicated brain regions, but evidence for spatial organization in respect to cardiac and pulmonary projections may help to discover this by a process of elimination (Aristovich et al., 2021).

### Treatment-Resistant Depression

Major depressive disorder (MDD), also known as clinical depression, is a psychological disorder in which an individual experiences consistent and persistent low mood for at least 2 weeks (Otte et al., 2016). Around 30% of MDD patients have treatment-resistant depression, usually defined as MDD that does not respond to two distinct courses of anti-depressant medication. VNS was approved for treatment-resistant depression in 2005, with over 4,000 patients currently undergoing this treatment (Otte et al., 2016).

### Inflammation

The VN serves as an important communication link between the immune system and brain. VNS has proven successful in treating disorders involving local and systemic inflammatory response due to its anti-inflammatory effects (Rosas-Ballina et al., 2011; Vida et al., 2011). VNS achieves these effects via activation of two major pathways: the cholinergic anti-inflammatory pathway (CAIP) and hypothalamic-pituitary-adrenal (HPA) axis (Hoffmann et al., 2012). CAIP involves release of acetylcholine (ACh) from VN efferents in the celiac mesenteric ganglia, which acts on post-synaptic α-7-nicotinic ACh receptors of the splenic nerve leading to the release of noradrenaline in the spleen, which dampens pro-inflammatory cytokine production by macrophages (Rosas-Ballina et al., 2011; Vida et al., 2011). Activation of HPA axis is attributable to vagal afferent fibers projecting to the nucleus of the solitary tract (NTS) in the brainstem. Stimulation of vagal afferents activates the adrenergic projections from NTS to the hypothalamus, release of corticotropin-releasing hormone and production of adrenocorticotropic hormone by the pituitary gland with an ultimate effect on the adrenal cortex and increased secretion of glucocorticoids (Hoffmann et al., 2012).

### Asthma and Chronic Obstructive Pulmonary Disease

Asthma and chronic obstructive pulmonary disease (COPD) are respiratory illnesses involving potentially life-threatening airway obstruction. This can be caused by enhanced parasympathetic activity, which results in airway smooth muscle contraction, increased mucus secretion and vasodilation in pulmonary vessels. This activity is also the dominant component of oedema in lung inflammation. High voltage VNS resulting in stimulation of vagal efferents in the lungs is ill-advised, as it could lead to bronchospasm as a side effect. However, low voltage VNS has been shown to preferentially activate vagal afferents and cause bronchodilation due to systemic increase of catecholamines via activation of the HPA axis (Hoffmann et al., 2012).

## Selective VNS

### Paradigms of VNS

A number of research groups have demonstrated that it is possible to achieve functionally specific effects from sVNS by selectively targeting and modulating organ function in various animal models and human patients (Pečlin et al., 2009; Plachta et al., 2014; Aristovich et al., 2021). sVNS results in similar or improved therapeutic effects compared to nsVNS, whereas side effects are reduced (Plachta et al., 2014). Hence, optimization of sVNS has become an important endeavor in nerve stimulation research.

Several major sVNS paradigms have been developed: fiber-selective stimulation, spatially selective stimulation, anodal block, kilohertz electrical stimulation (KES) block and neural titration. Across these paradigms, the development of sVNS techniques has typically focused on optimizing the shape of the stimulation pulse, the geometry of the electrode array and the stimulation protocol. In the following section, we provide an overview of the methods, effects and recent advances of the major sVNS paradigms.

### Existing sVNS Techniques

The most basic approach to sVNS is to identify which branch of the nerve projects to the organ(s) of interest, and then stimulate that branch. This method has been used since at least 1992, when Furukawa and Okada (1992) demonstrated responses in the gallbladder of a dog from stimulation of the whole gastric branch (Furukawa and Okada, 1992). However, this method is incompatible with established surgical procedures. These are optimized for implantation of cuff arrays around the cervical VN with minimal complications (Ben-Menachem, 2002). Thus, it is more desirable to achieve sVNS through optimized stimulation of the cervical VN.

One potential approach for achieving selectivity at the cervical level is to surgically tease apart the VN and apply stimulation solely to particular fascicles. This could be done with a microchannel array (Lancashire et al., 2016). However, the functional anatomy of the VN is not characterized well enough. It would also be highly invasive, risking severe irreversible nerve damage. For these reasons, microchannel arrays are not widely used in humans, and less invasive sVNS procedures are desired.

Besides transcutaneous stimulation – which is unlikely to produce selective effects due to current spreading as it passes through the skin and connective tissue – the least invasive practice uses a cuff array that wraps around the nerve (Chapman et al., 2018). With such arrays, two main paradigms for sVNS have been demonstrated:

(1) fiber-selective stimulation: exploits the different activation thresholds of VN fibers to separately activate selected fiber type.
(2) spatially selective stimulation: application of electrical stimulus to specific area of the nerve cross-section to only activate selected fascicles.

Both approaches have recently been demonstrated (Plachta et al., 2014; Dali et al., 2018), with the fiber-selective approach proving more popular (see Table 1). However, evidence for spatial organization in the VN, combined with difficulties in avoiding the activation of larger fibers, has motivated the search for a spatially selective paradigm (Plachta et al., 2014). One should also bear in mind that it is not possible to completely separate these two paradigms, since protocols that aim at fiber-selectivity usually involve some degree of spatial-selectivity and vice versa (Vuckovic et al., 2008).

**TABLE 1:** Major recent papers on sVNS in chronological order.

**Publication**	**sVNS Method(s)**	**Outcomes**	**Pulse parameters**	**Electrode geometry**
Tosato et al., 2007. Quasi-trapezoidal pulses to selectively block the activation of intrinsic laryngeal muscles during vagal nerve stimulation,” *J. Neur. Eng*. 4. 3. 205–12.	Anodal block for fiber-selective control of HR in pig.	Success lowering HR laryngeal side effects reduced by 77%.	Quasi-trapezoidal pulse. Flat phase 0.6 ms and exponentially decaying phase 2.4 ms; maximal response at 5–15 mA with QT pulses, 0.5–20 Hz, Figure 2F.	3.4 mm inner diameter cuffs with 1 mm wide platinum rings, 4 mm spacing in between rings Figure 3B.
Vuckovic et al., 2008. A comparative study of three techniques for diameter selective fiber activation in the vagal nerve: anodal block, depolarizing prepulses and slowly rising pulses. *J. Neur. Eng.* 5. 3. 275–86.	Compare anodal block, depolarizing pre-pulses, slowly rising pulses; selectively activate smaller fibers in pig.	60–100% reduction in Aβ fiber activity with anodal block. Up to 90% reduction with depolarizing pre-pulses. Up to 60% with slowly rising pulses.	Anodal block: quasi-trapezoidal pulse with 0.4–1 ms flat period and up to 1 ms exponentially decaying phase. 4–12 mA. Max 30 Hz, Figure 2B. Depolarizing pre-pulse: 0.2–0.8 ms low amplitude pulse (highest excitation threshold determined experimentally) followed by 0.2–0.6 ms higher amplitude pulse (2–6 mA). Max 105 Hz, Figure 2C. Slowly rising pulses: 1–5 ms exponentially or hyperbolically rising curve followed by 0–0.1 ms flat period. 2–6 mA. Max 28 Hz, Figure 2D.	Split cylinder cuff electrodes. Tripole with 3 mm separation between contacts used for stim.
Rozman and Peclin, 2008. Selective stimulation of autonomic nerves and recording of electroneurograms in a canine model. *Artificial Organs.* 32. 8. 592–6.	Fiber-selective stim with electrodes in varying positions around nerve circumference control HR and RR in dog.	Successful selective modulation of HR and RR.	Current, biphasic, charge balanced quasi-trapezoidal pulse as in Peclin and Rozman (2009), see above, Figure 2F.	39 rectangular electrodes arranged in a matrix of 9 parallel groups, with the stim section 11 groups of 3 electrodes in the middle of the matrix, and two blocking sections with 11 electrodes each positioned bilaterally to the stim section, Figure 3F.
Peclin and Rozman, 2009. A model of selective left VNS and recording in a man. *IFMBE*. 1628–31.	Fiber-selective stim to control HR in humans.	Successful reduction of HR preferential activation of B fibers over A fibers.	Current, biphasic, charged- balanced quasi-trapezoidal pulse; cathodic with approx 1 mA square leading edge, 0.3 ms plateau and exponentially decaying phase of 0.3 ms; anodic rectangular pulse of low magnitude Figure 2F.	39 rectangular electrodes with thirteen circumferential groups of 3 electrodes. 0.6 × 1 mm. Inner diameter of cuff 2.5 mm, length 20 mm. [Details given in Rozman et al. (1993)] Figure 3F.
Ordelman et al., 2013. Selectivity for Specific cardiovascular effects of Vagal nerve Stimulation with a multi-contact electrode cuff. *IEEE Trans. Neural Syst. Rehab. Eng*. 21. 1.	Spatially selective stim with a muti-contact cuff in pig.	Increased efficacy in cardiac modulation compared to nsVNS (greater number of cardiac parameters significantly altered by stim).	Biphasic pulses. Second pulse has exponential shape, 1st phase pulse width 0.3 ms, 1–10 mA, 10–50 Hz. Burst stim maintained up to 60 s, Figures 2A,B.	One config has rings, 15 mm long with 3 circular electrodes, interelectrode distance 4 mm. Surface area of each 2 mm^2^. Spacing of electrodes at 90-degree intervals, Figure 3B.
Plachta et al., 2013. BaroLoop: using a multichannel cuff electrode and selective stimulation to reduce blood pressure. *Conf. Proc. IEEE Eng. Med. Biol. Soc.* 755–8.	Demonstration of system for BP control via spatially selective stim. Data from rats.	Successful control of BP with “almost no side effects.”	Biphasic rectangular pulses, adjusted for charge balance. Tripole which shows baroreceptive activity located; center electrode of this tripole is cathode against two large ring electrodes. 200 pulses per stim 30–50 Hz, 0.3–1 mA, inter-stimulus interval 10 s Figure 2A.	24 electrodes, arranged in 8 tripoles around the cuff perimeter with 45 degree spacing. Cuff length 12 mm, diameter 0.8 mm. Distance between cross-sectional electrodes 2 mm Figure 3D.
Plachta et al., 2014. Blood pressure control with selective vagal nerve stimulation and minimal side effects. *J. Neur. Eng.*. 11. 036011.	Spatially selective tripolar stim in rats control BP without altering HR.	Significant reduction in BP with no bradypnea and less than 25% reduction in HR.	Current controlled, charge balanced, rectangular pulses 200 pulses per stim. Interval at least 10 s between stim 30–50 Hz, 0.3–1 mA, pulse width 0.1–0.5 ms, Figure 2A.	Same as in Plachta et al., 2013 (see above).
Pečlin and Rozman, 2014. Alternative paradigm of selective VN stimulation tested on an isolated porcine VN. *The Scientific World Journal*. 310283.	Fiber-selective stim. Experiments in pigs. Demonstration of “quasi-trapezoidal” pulse shape.	Limited fiber-selective VNS was achieved, with increased A fiber activation and decreased B fiber activation.	Current, biphasic, charged- balanced quasi-trapezoidal pulse; cathodic with approx 1 mA square leading edge, 0.3 ms plateau and exponentially decaying phase of 0.3 ms; anodic rectangular pulse of low magnitude Figure 2F.	99 rectangular electrodes arranged in a matrix of 9 parallel groups, with the stim section 11 groups of 3 electrodes in the middle of the matrix, and two blocking sections with 11 electrodes each positioned bilaterally to the stim section Figure 3G.
Qing et al., 2015. Burst-modulated waveforms optimize electrical stimuli for charge efficiency and fiber selectivity. *IEEE Trans. Neural Syst. Rehab. Eng*. 23. 6. 936–45.	Bursts of small rectangular pulses for spatially selective stim.	C fibers kept above 50% activation with activation of A fibers reduced 11% compared to nsVNS.	Charge balanced, cathode leading, alternating monophasic rectangular waveforms or burst waveforms 10 s stim followed by 10 s recovery; pulse width 40–200 μs, Max amplitude 1.5 mA, 10–20 Hz Figure 2A,E.	Leads spaced 1 mm apart with contact area 0.011 cm^2^ for each lead.
Patel and Butera, 2015. Differential fiber-specific block of nerve conduction in mammalian peripheral nerves using kilohertz electrical stimulation. *J. Neurophysiol.* 113. 10. 3923–9.	Fiber-selective stim with KES in rats.	Able to selectively block the fast and slow components of the compound action potential.	Supramaximal cathode-first biphasic pulses 5 V, 0.2 ms KES block stimulus is continuous sinuosoid, 50–70 kHz.	Tripolar, longitudinally slit cuff. 0.75 mm between contacts, cuff diameter for 1–1.2 mm and length 3 mm, Figure 3B.
Plachta et al., 2016. Effect of cardiac-cycle synchronized selective vagal stimulation on heart rate and blood pressure in rats. *Advances in Therapy*. 33. 7. 1246–61.	Spatially selective stim using pulsatile stimulus synchronized to cardiac cycle. Experiments in rats.	Able to reduce BP and keep it lower without significant bradycardia.	Biphasic rectangular pulses, 100 pulses in three sets 30–50 Hz, 0.2–0.9 mA, 0.2–0.9 ms pulse width Figure 2A.	24 electrodes, arranged in 8 tripoles around the cuff perimeter with 45 degree spacing. Cuff length 12 mm, diameter 0.8 mm. Distance between cross-sectional electrodes 2 mm, Figure 3D.
Yoo et al., 2016. Modulation of heart rate by temporally patterned VN stimulation in the anesthetized dog. *Physiol. Rep.* 4:12689.	Fiber-selective stim in dogs.	Able to selectively modulate HR and laryngeal EMG. Laryngeal side effects during cardiac modulation reduced 50% compared to nsVNS.	1 s inter-burst interval, 20 s pulse train, 2–20 pulses per burst, pulse width 0.3 ms, frequency 10–50 Hz thresholds a fibers 0.08 mA, fast B 1.5 mA, slow B 4.4 mA, Figure 2A.	Bipolar, helical electrode Figure 3A.
Patel et al., 2017. Kilohertz frequency nerve block enhances anti-inflammatory effects of VN stimulation. *Nature Scientific Reports.* 7. 39810.	KES for virtual vagotomy, directionally specific block.	Successful unidirectional block in most cases, although block was sometimes incomplete.	Biphasic constant current pulses 1 mApp, 0.4 ms pulse width, 1 Hz KES at 40 kHz, 1.5–2 mA peak.	Custom, bipolar electrodes, stainless steel wire threaded through silicone tubing and spot welded to Pt-Ir contact pads Figure 3C.
Dali et al., 2018. Model based optimal multipolar stimulation without *a priori* knowledge of nerve structure: application to VN stimulation. *J. Neur. Eng*. 15.4. 046018.	Spatially selective stim for cardiac modulation. Experiments in sheep.	62% reduction in side effects compared to nsVNS.	Rectangular pulses acute tests: on 60 s, off 30 s; pulse width 240 μs, 25.6 Hz frequency; 4 pulses per cardiac cycle. Implant-explant: on 16 s, off 44 s; 25.6 Hz frequency; Pulse width 300 μs 0.2–1.5 mA (anesthesia), 1–3 mA (conscious) chronic: on 30 s, off 30 s; 25.6 Hz frequency pulse width 300 μs, Figure 2A.	Modeling of different geometries (ring, tripolar longitudinal ring (TLR), transverse tripolar (TT), transverse tripolar ring (TTR) with cathode at 0, 90, 180, and 270 degrees around the circumference Figure 3H.
McAllen et al., 2018. Calibration of thresholds for functional engagement of vagal A–C fiber groups *in vivo*. *Bioelectronic Medicine*. 1. 1. 21–27.	Fiber-selective stim. Experiments in rats.	Monitoring HR and RR while changing stimulating modality allowed for thresholds of different fiber types to be found.	Constant voltage square pulses pulse width 0.1 ms 1–2 Hz Figure 2A.	2 Electrodes, details of geometry not given.
Dali et al., 2019. Comparison of the efficiency of chopped and non-rectangular electrical stimulus waveforms in activating small VN fibers. *J. Neurosci. Methods.* 320. 1–8.	Fiber-selective stim. Modeling and then experiments in pigs.	Ramp-shaped pulse and sine-wave shaped chopped pulse good for targeting smaller fibers.	Chopped pulses, rectangular and ramp rectangular, ramp, quarter-sine: pulse width 350 μs chopped quarter-sine: 325 μs or 1 ms. Amplitude corresponded to charge of 1.5 nC. Frequency 2 Hz. 6 pulses with 1 s inter-pulse interval. Figure 2E.	Two rows of Pt-Ir electrodes with a diameter of 3 mm. Rows shorted together to form a bipolar ring. Figure 3C.
Aristovich et al. (2021). Model-based geometrical optimisation and in vivo validation of a spatially selective multielectrode cuff array for vagus nerve neuromodulation. *J. Neuroscience Methods.* 352. 109079.	Spatially selective stim. Modeling with FEM and experiments in sheep.	Can selectively lower RR by up to 90% without significant change in HR, and lower HR up to 27% without significant change in RR.	30 s stim, 30 s recovery square, biphasic (positive first) constant current temporal waveform with balanced current source pulse width 100 and 50 μs per phase, no interpulse interval 20 Hz frequency, 450–550 μA optimal for RR change without HR change Figure 3A.	Modeling of various geometries. optimal array fabricated with 14 longitudinal electrode pairs, 3 mm apart, width 0.4 mm and 0.35 mm interelectrode circumference distance, 3 mm length Figure 3E.

*Summary of results, methods, pulse parameters and electrode geometry. RR, respiratory rate. HR, heart rate. BP, blood pressure. KES, kilohertz electrical stimulation.*

**FIGURE 2:**
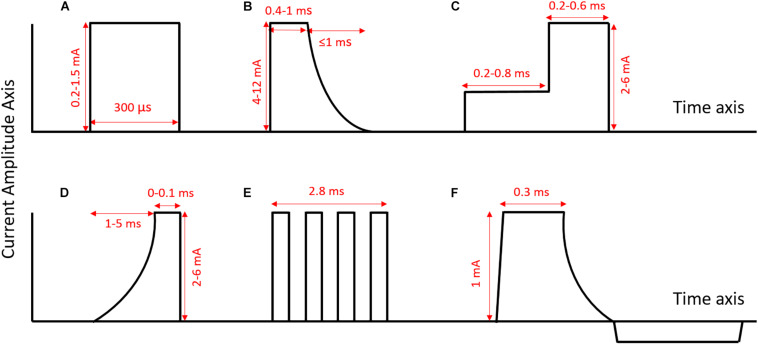
Examples of different pulse shapes used in sVNS (axes not to scale). **(A)** Normal rectangular pulse [as used in Dali et al. (2018)]. **(B)** Anodal block pulse [as used in Vuckovic et al. (2008)]. **(C)** Depolarizing pre-pulse [as used in Vuckovic et al. (2008)]. **(D)** Slowly rising pulse [as used in Vuckovic et al. (2008)]. **(E)** Chopped pulse [as used in Dali et al. (2019)]. **(F)** Quasi-trapezoidal pulse [as used in Pečlin and Rozman (2014)]. Rectangular pulses are the standard pulse shape used in the nerve stimulation. They are easy to generate, and their symmetry makes it easier to ensure charge balance. However, pulses with more unusual shapes, such as chopped pulses or pulses with ramped parts, allow for the delivery of current throughout the pulse to be adapted to specific applications.

With a cuff array, it is also possible to implement blocking of nerve impulse propagation (both selectively and non-selectively). Kilohertz electrical stimulation block (KES block) is a type of blocking that is designed to ensure impulses only travel in one direction along the VN; this ensures a certain degree of functional selectivity, akin to that achieved by vagotomy (Patel and Butera, 2018). KES can also be used as a technique to achieve fiber-selective stimulation (Vuckovic et al., 2008), as can the more common form of nerve blocking, anodal block. Anodal block occurs when the anode (positive terminal) of a pair of electrodes causes hyperpolarization in the section of the nerve below it (Figure 2B; Vuckovic et al., 2008); hyperpolarizing axons (bringing them to a negative potential) closes voltage-gated sodium channels in the plasma membrane, preventing an action potential from being generated. The mechanisms of KES are still being investigated, although it is believed that KES inactivates sodium channels through excessive depolarization of the nerve (Kilgore and Bhadra, 2004). These forms of the block are often imperfect; realistically, they are more likely to achieve a partial block than a full directional selectivity.

Neural titration, introduced by Ardell and colleagues (Ardell et al., 2015, 2017), relies on the establishment of a dynamic equilibrium (neural fulcrum) that cancels out side effects (in their case focusing on bradycardia). Fibers that elicit bradycardia (vagal efferents) and fibers that elicit tachycardia (afferents) were activated at the same time, and stimulation amplitude was adjusted until the effects of the two fiber-types were perfectly balanced (Ardell et al., 2017).

### Fiber-Selective VNS

Most of the recent research in sVNS has focused on applications for cardiovascular disease (see Table 1). Whereas VNS for epilepsy and MDD primarily requires activation of larger fibers (A and B types), cardiac neuromodulation primarily requires activation of smaller fibers (B and C types) (Dali et al., 2018). Since the threshold for activation of smaller fibers is at a higher current amplitude (Bawa et al., 2014), cardiovascular VNS would be expected to engender more severe side effects than VNS for epilepsy and MDD. Thus, several research groups have focused on developing fiber-selective VNS for cardiovascular applications.

**FIGURE 3:**
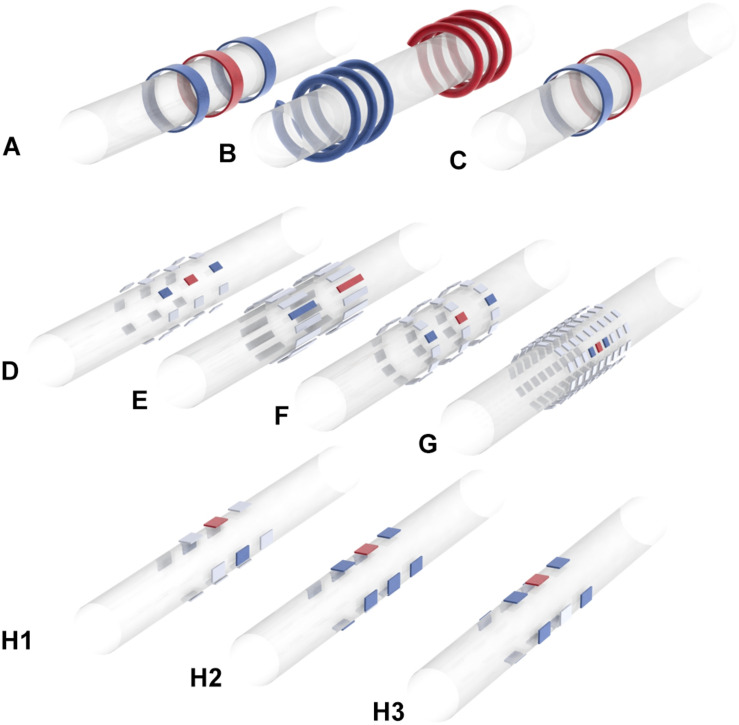
Electrode array geometries and stimulation patterns used in major sVNS studies. **(A)** Yoo et al. (2016). **(B)** Ordelman et al. (2013), Patel and Butera (2015), Tosato et al. (2007). **(C)** Pelot and Grill (2020), Patel et al. (2017), Dali et al. (2019). **(D)** Plachta et al. (2013, 2014, 2016), Gierthmuehlen and Plachta (2016). **(E)** Aristovich et al. (2021). **(F)** Peclin and Rozman (2009). **(G)** Pečlin and Rozman (2014). **(H)** Dali et al. (2018) [**H1**: transverse tripolar (TT), **H2**: transverse tripolar ring (TTR), **H3**: tripolar longitudinal ring (TLR)]. Blue = anode, red = cathode, gray = unused. Researchers have used a range of different electrode array geometries for sVNS. Some have used rings or helical electrodes; recently there is a move toward smaller rectangular contacts that can be placed at different positions around the nerve circumference. These contacts are often arranged to allow for bipolar or tripolar stimulation.

Tosato et al. (2007) explored the selective control of HR in a porcine model, using anodal block to prevent the activation of larger fibers (Figures 2B, 3A). They were able to successfully lower HR while reducing laryngeal side effects by up to 77% (Tosato et al., 2007). They compared three different methods for achieving selective activation of cardiac vagal fibers: depolarizing pre-pulses, slowly rising pulses and anodal block (Vuckovic et al., 2008). A depolarizing pre-pulse (Figure 2C) is a small stimulus, which arrives just before the main stimulatory pulse with an amplitude just below the excitation threshold of the largest fibers; this pre-pulse raises the excitation threshold of those fibers through sodium channel inactivation (although it may also lead to anodal break excitation when it ends) (Vuckovic et al., 2008). Slowly rising pulses (Figure 2D) have an initial curved ramp, which reverses recruitment order by inactivating sodium channels and exploiting variations in the spatial distribution of membrane potential between fiber types (Hennings et al., 2005). Anodal block completely prevented activation of Aβ fibers in two out of five pigs, and reduced Aβ activity by 60–90% in the other three (Vuckovic et al., 2008). The large diameter of the VN in pigs necessitated high-amplitude current (up to 10 mA) to implement the block. This may have led to excitation of fibers at the edge of the nerve, hence preventing full block (Vuckovic et al., 2008). Depolarizing pre-pulses achieved up to a 90% reduction in Aβ activity; slowly rising pulses achieved up to 60%. This was determined by observing the reduction in size of the Aβ component of the compound action potential via electroneurogram. The authors endorse depolarizing pre-pulses as their preferred method, being the only one of the three approaches that was effective with safe levels of charge-injection (Vuckovic et al., 2008). It should be noted that whether a pulse can be achieved with safe charge injection depends to some extent on electrode geometry, and it may also vary between species.

Ahmed et al. (2020) have demonstrated that anodal block is capable of eliciting a significant degree of directional selectivity in the rat VN, although efficacy was inconsistent. Swapping the orientation of the cathode and anode was associated with preferential activation of efferent or afferent fibers, as shown by differential effects on breathing or HR, respectively. In 3 out of 17 rats, however, the opposite effect of electrode polarity was observed; the authors attribute this to anatomical differences between rats, specifically the position of the aortic depressor nerve relative to the main trunk of the VN (Ahmed et al., 2020). The possible effects of anatomical variation must be noted when attempting to translate anodal block to human patients.

Peclin and Rozman (2009) were the first to demonstrate fiber-selective VNS in humans (Pečlin et al., 2009; Peclin and Rozman, 2009), following earlier work in a canine model (Rozman and Peclin, 2008). In the dog, they demonstrated selective control of HR and respiration rate by stimulating with sets of three electrodes at different positions around the nerve circumference (Figures 3F,G; Rozman and Peclin, 2008). They then applied the same technique to two human patients, reducing the HR while preferentially activating B over A fibers (Pečlin et al., 2009). Their method involves the use of “quasi-trapezoidal” pulses (Figure 2F), which activate A and B fibers during their square cathodal phase, before blocking A fibers during an exponentially decaying anodal phase (Pečlin and Rozman, 2014). However, in humans, this work has only been published in the form of conference abstracts and has not been peer-reviewed.

Qing et al. (2015), working in a rat model, achieved fiber-selective stimulation through the use of “chopped pulses,” replacing normal rectangular waves with repeated bursts of smaller rectangular waves whose width, interval and number can be modulated (Figure 2E; Qing et al., 2015). The earlier pulses in the burst caused inactivation of sodium channels, primarily in large fibers, allowing smaller fibers to be preferentially excited by the later pulses. Chopped pulses are easier to produce with a normal stimulator than quasi-trapezoidal waves, and avoid the need that pre-pulses have for a precise determination of excitation threshold (Qing et al., 2015). A and C fiber activation were identified by Qing and colleagues from two separate peaks in the compound nerve action potential, with the height of the peak taken as the degree of activation. Chopped pulses and normal rectangular pulses were compared at 50% of the charge required to elicit the maximum C fiber response. At this charge level, chopped pulses where able to maintain C fibers at 50% activation while reducing activation of A fibers by 11% compared to stimulation with normal rectangular waves (Qing et al., 2015). While 11% is a significant reduction, Qing and colleagues did not investigate if it was sufficient to significantly mitigate side effects.

Yoo et al. (2016) applied a similar method in dogs, demonstrating successful HR modulation while reducing laryngeal side effects by 50% compared to nsVNS (Yoo et al., 2016). Here, the extent of laryngeal side effects was indicated by the amplitude of laryngeal electromyogram signals. The authors claim that chopped pulses displayed comparable or superior efficacy to nsVNS. However, this is not true if stimulation amplitude is held constant: their data shows that nsVNS elicits a greater reduction in HR than chopped pulses at the same frequencies when the amplitude is above the bradycardia threshold (Yoo et al., 2016); thus, selective stimulation here entailed lower efficacy.

Dali et al. (2019) compared chopped to continuous pulses, while also varying the overall pulse shape, creating a chopped-ramp (linearly increasing amplitude of each pulse within the train) and a chopped quarter-sine (amplitude of pulses within the train follows part of a sine wave). They focused on afferent gastric fibers, stimulating them distally and recording proximally at individual afferent B fibers. The threshold charge required to activate the B fibers was 19% lower for a ramp pulse than a rectangular pulse, and 15% lower for a quarter-sine pulse than a rectangular. The continuous ramp was the most energy efficient pulse shape (Dali et al., 2019).

### Spatially Selective VNS

The first study to focus on spatially selective VNS was Ordelman et al. (2013), who suggested this approach as a solution to the difficulties faced by Tosato et al. (2007) in achieving full block of the targeted fiber type (Ordelman et al., 2013). Full block of a certain fiber type may be difficult to achieve if the fibers have highly varying cross-sectional position within the nerve. Ordelman and colleagues, working with a multi-contact cuff (Figure 3B), achieved almost double the efficacy in cardiac modulation compared to conventional VNS in pigs; 20–60 s after stimulation, HR measured by RR interval was reduced by 10% with nsVNS, and 18% with sVNS. The variance in HR reduction was quite high, however, especially with sVNS (Ordelman et al., 2013).

The same year, Plachta and colleagues presented their BaroLoop spatially selective VNS system (Figure 3C), demonstrating successful modulation of blood pressure (BP) in the rat; BP was reduced to 60% of the baseline value with no significant bradycardia or bradypnea (Plachta et al., 2013). It was also possible to achieve a permanent reduction in baseline BP if treatment was applied chronically. However, this study did not compare the efficacy of sVNS to nsVNS, but only compared an optimal selective paradigm to a non-optimal selective one. Nonetheless, it did provide evidence that fibers specific to BP modulation are localized to one side of the VN, since stimulation at the side directly opposite elicited bradycardia with no reduction in BP (Plachta et al., 2013).

Plachta and colleagues, who have been the main research group developing spatially selective techniques, presented similar results in 2014 (Plachta et al., 2014). In rats, they first localized the fibers responsible for BP control by measuring the response for different tripoles, before using sVNS to lower BP up to 40%. They observed no significant bradypnea and maximum 25% bradycardia, but did not prevent laryngeal side effects. In subsequent studies, they showed that the BP-reducing effects of their technique were attenuated but still significant in the presence of several major anti-hypertensive drugs (Gierthmuehlen and Plachta, 2016; Gierthmuehlen et al., 2016). However, stimulation-induced apnea was significantly increased in the presence of metoprolol (Gierthmuehlen and Plachta, 2016).

Dali et al. (2018) attempted to translate spatially selective VNS to the sheep (Dali et al., 2018). First, they conducted a modeling study with a finite element model of a nerve in order to optimize their stimulation parameters. The nerve model had 22 fascicles and was derived from cross-sectional images of a sheep VN. The Laplace equation was solved on the FEM in COMSOL, and then the optimal stimulation parameters were determined via a cost function that maximizes efficiency, selectivity and sensitivity to current amplitude. It was found that a configuration which the authors called “transverse tripolar” (TT) provided the best selectivity (Figure 3H1). However, when implementing sVNS *in vivo*, another configuration called “transverse tripolar + ring” (TTR) provided an optimal balance between selectivity and efficiency (Figure 3H2). Both were superior to the “tripolar longitudinal ring” (TLR) (Figure 3H3) and to the “ring” (nsVNS). With TTR, they were able to control HR while reducing side effects by 62% compared to nsVNS. However, the authors do not specify which particular side effects were included in their side effects index. It is also not clear whether any of the selective configurations was as effective as nsVNS.

Aristovich et al. (2021) aimed to further develop spatially selective VNS through optimization of the geometrical parameters in a sheep model (Aristovich et al., 2021). Initial computer modeling suggested the best geometry was a symmetrical arrangement with electrodes at the same position around the circumference (Figure 3E). This geometry was tested in twelve sheep, where it was possible to selectively reduce respiration rate by 90% without significant bradycardia and HR by 27% without significant bradypnea. Comparing the percentage HR and respiration rate changes across different stimulation modalities also indicated marked spatial-functional organization of the sheep VN. Laryngeal side effects were not considered by Aristovich et al. (2021).

The spatial selectivity has a limited value with respect to avoidance of the therapy-limiting side effects as they are mediated by Aα motor fibers and Aβ sensory fibers which have much lower activation thresholds (Ardell et al., 2017). For instance, it is possible that the laryngeal motor fibers would be activated during the therapeutic spatially selective stimulation even if they are located spatially on the other side of the nerve. This technique, however, can potentially be combined with directional selectivity such as anodal block (Ahmed et al., 2020) to at least partially overcome the stated limitations.

### Kilohertz Electrical Stimulation Block

KES block is a technique in which an electrical stimulus of at least 5 kHz is applied to a nerve to inhibit action potential propagation (Patel and Butera, 2018). The technique was first demonstrated in the sciatic nerve of a frog, before being applied to the VN by Patel and Butera (2015). Traditionally, KES has aimed to block the entire nerve, providing only directional selectivity, although the work by Patel and Butera (2015) has shown that this kind of blocking can also achieve fiber-selective stimulation (Patel and Butera, 2015). In practice, it is rare for such directionally selective techniques to achieve a complete block even when this is their aim. It is also not clear what practical advancements KES seeks to make over anodal block.

Patel et al. (2017) used KES of the whole nerve to achieve directionally selective stimulation (Patel et al., 2017). In the rat cervical VN, they were able to selectively activate the efferent pathways while inhibiting transmission along the afferent ones (and hence simulate a vagotomy). Their aim was to improve anti-inflammatory VNS for the treatment of rheumatoid arthritis. Compared to nsVNS, full block via KES delivered an improved anti-inflammatory effect. However, when KES only achieved partial block, systemic inflammation was worse. KES also sometimes lead to severe nerve damage if parameters were not carefully chosen (Patel et al., 2017).

### Neural Titration

Neural titration relies on antagonistic mechanisms within vagal control of cardiac function, as discovered by Ardell and colleagues (Ardell et al., 2015). Afferent and efferent fibers have opposite effects on the modulation of HR. Rather than attempt to avoid activation of fibers causing bradycardia, afferent and efferent fibers can be simultaneously activated to precisely the extent required for the bradycardic effects of efferent fibers to cancel out the tachycardic effects of afferent fibers (Ardell et al., 2015). Ardell and colleagues call this dynamic equilibrium “the neural fulcrum”; it is defined as the current amplitude just below that at which bradycardia is reliably evoked (bradycardia defined as a 5% decrease in HR in three consecutive stimulation sessions) (Ardell et al., 2017). Ardell and colleagues assessed the efficacy of this technique in dogs (Ardell et al., 2015). Stimulation parameters were varied to find the neural fulcrum, which remained stable for at least 14 months. However, their method had limitations. Thirty days of sessions were required to find optimal stimulation parameters for each dog, which is impractical for clinical use (although machine learning could potentially accelerate this). The dogs used in this study were healthy animals and not canine models of heart failure (Ardell et al., 2017). It is possible then that heart disease could change the behavior of the peripheral nervous system (PNS) in ways that would make neural titration more difficult to implement. Also, the controlled environment of the study likely increased the reliability of the neural titration by avoiding large changes in the animals’ environment and individual physiological conditions; it is possible that a patient under non-controlled conditions may experience functional changes in their PNS that would shift the neural fulcrum on a short timescale.

## Conclusion

The endeavor to develop sVNS has resulted in a range of promising techniques. Most studies have focused on fiber-selective stimulation, in some cases achieving an impressive reduction in laryngeal side effects (Tosato et al., 2007; Vuckovic et al., 2008). Anodal block and depolarizing pre-pulses have demonstrated the strongest mitigating effects (Tosato et al., 2007; Vuckovic et al., 2008), while chopped pulses and slowly rising pulses have been less effective (Vuckovic et al., 2008; Qing et al., 2015). More recent studies investigating the possibility of spatially selective VNS yelded more promising results (Plachta et al., 2014; Dali et al., 2018; Aristovich et al., 2021).

Fiber-selective sVNS is the only form of sVNS demonstrated in human patients (Pečlin et al., 2009), although peer-reviewed human studies are yet to emerge. Furthermore, studies on fiber-selective VNS have tended to focus on acute applications. Since the major therapeutical effects of VNS are seen chronically (Krahl, 2012), translation of fiber-selective VNS to a clinical setting would require validation of the technique chronically and with a large number of patients (in contrast to the low n numbers in animal studies). It is crucial to establish whether the efficacy of fiber-selective techniques varies significantly between patients. It is also necessary in such studies to ensure that fiber-selective techniques do not compromise electrochemical safety (an issue that has not been addressed in the sVNS literature to date).

Studies focusing on anodal block typically do not attempt to use neuromuscular blockade or nerve transection to isolate the pathway mediating respiratory side effects. This means that these studies are unable to assess the effects of possible off-target field escape (current leaking through the insulation of the stimulation apparatus). Accounting for this possibility is complicated by the fact that there may be multiple pathways whose activation can trigger respiratory side effects (such as Hering-Breuer reflex via activation of Aδ fibers, closure of glottis due to activation of Aα fibers, or a cough reflex due to activation of Aβ fibers).

Unlike fiber-selective sVNS, spatially selective sVNS takes into account evidence for a somatotopic arrangement of fibers inside the VN (Aristovich et al., 2021). Spatially selective VNS provides more precise targeting than fiber-selective VNS, and has been used successfully to elicit organ-specific responses (Aristovich et al., 2021). It has demonstrated comparable mitigation of side effects to fiber-selective VNS (Plachta et al., 2014; Dali et al., 2018), as well as increased efficacy in cardiac modulation (Ordelman et al., 2013). While studies cited here have examined the relationship between spatially selective VNS and the strength of side effects affecting the cardiovascular and respiratory system, these studies have not investigated how stimulation at different positions around the vagal circumference affects the activation of laryngeal muscles. This is a key oversight that must be addressed in future studies, since laryngeal side effects are the most common side effects experienced by patients receiving VNS. Research into spatially selective VNS also generates information on VN anatomy required for optimization of stimulation parameters (Plachta et al., 2014; Aristovich et al., 2021).

Achieving a better understanding of VN anatomy is the most important step toward improving sVNS techniques. While great progress has been made in this area (Thompson et al., 2019, 2020; Settell et al., 2020), it is still not established definitively whether fibers in the VN are arranged by fiber-type or by innervated organ, with current evidence inconclusive. If VN fibers are arranged both by fiber-type and somatotopically, then it would be necessary to develop hybrid sVNS techniques that are both fiber-selective and spatially selective.

All studies investigating the potential of sVNS to mitigate side effects must give careful consideration to the anesthetic used. Isoflurane, as used by Ordelman et al. (2013) and by Plachta et al. (2014), has a depressive effect on the PNS and on baroreflex in particular. Thus, side effects that would arise during vagal stimulation in an awake animal may be dampened or absent in an animal anesthetized with isoflurane. Isoflurane could also reduce the efficacy of VNS compared to an awake animal. Anesthetics such as urethane, α-chloralose or ketamine interfere less with peripheral nerve activity and should be used instead of isoflurane in VNS experiments.

Typically, studies that have assessed laryngeal side effects during VNS have focused on measuring EMG of a single deep neck muscle. This does not account for the possibility of current leakage through the apparatus insulation. It has also not been established whether a reduction in laryngeal EMG is associated with increased patient tolerance to the treatment. Patient tolerance must be assessed to allow for clinical translation. Moreover, since motor tolerability varies over time due to habituation, patient tolerance of laryngeal side effects during sVNS must be evaluated chronically.

How the long-term habituation may affect the efficacy of sVNS techniques is also yet to be determined. To date, all studies on sVNS in animals have been conducted acutely in anesthetized subjects. Various factors are arising after surgery that could impact the efficacy of sVNS. The formation of scar tissue could change the distance between the array and the target site as well as the pattern of current flow. Neuroplasticity could alter the response profile of the nerve. The position of the neck and the balance of fluids in the body may also have a significant impact. The design of closed-loop sVNS systems may be useful in responding to this long-term variability (Ahmed et al., 2020).

It is necessary to expand the application of sVNS beyond cardiac and respiratory therapeutic modalities. At present, the only FDA-approved clinical uses of VNS are for focal epilepsy and treatment-resistant depression (O’reardon et al., 2006; Krahl and Clark, 2012), but sVNS has not been explored for either. Focal epilepsy in particular warrants especial attention, since it has demonstrated high efficacy and is in use in a large number of patients worldwide (Krahl and Clark, 2012). The use of VNS to mitigate inflammation also remains a promising endeavor (Kwan et al., 2016; Mastitskaya et al., 2021).

The development of better sVNS techniques has the potential to benefit hundreds of thousands of patients worldwide (Johnson and Wilson, 2018), but sVNS is still a small research area. The better understanding of VN anatomy, development of more precise stimulation techniques and optimized electrode array geometries would drive the progress of sVNS research and its translation into clinical practice.

## Author Contributions

KA and SM conceptualized and supervised the work. AF and SM wrote the manuscript. All authors contributed to the article and approved the submitted version.

## Conflict of Interest

The authors declare that the research was conducted in the absence of any commercial or financial relationships that could be construed as a potential conflict of interest.
